# Neural Habituation to Painful Stimuli Is Modulated by Dopamine: Evidence from a Pharmacological fMRI Study

**DOI:** 10.3389/fnhum.2017.00630

**Published:** 2017-12-21

**Authors:** Eva M. Bauch, Christina Andreou, Vanessa H. Rausch, Nico Bunzeck

**Affiliations:** ^1^Department of Systems Neuroscience, University Medical Center Hamburg-Eppendorf, Hamburg, Germany; ^2^Medical School Hamburg (MSH), University of Applied Science and Medical University, Hamburg, Germany; ^3^Center for Gender Research and Early Detection, University Psychiatric Clinics Basel, Basel, Switzerland; ^4^Department of Diagnostic and Interventional Radiology and Nuclear Medicine, University Medical Center Hamburg-Eppendorf, Hamburg, Germany; ^5^Institute of Psychology I, University of Lübeck, Lübeck, Germany

**Keywords:** pharmacological fMRI, EEG, pain, habituation, haloperidol

## Abstract

In constantly changing environments, it is crucial to adaptively respond to threatening events. In particular, painful stimuli are not only processed in terms of their absolute intensity, but also with respect to their context. While contextual pain processing can simply entail the repeated processing of information (i.e., habituation), it can, in a more complex form, be expressed through predictions of magnitude before the delivery of nociceptive information (i.e., adaptive coding). Here, we investigated the brain regions involved in the adaptation to nociceptive electrical stimulation as well as their link to dopaminergic neurotransmission (placebo/haloperidol). The main finding is that haloperidol changed the habituation to the absolute pain intensity over time. More precisely, in the placebo condition, activity in left postcentral gyrus and midcingulate cortex increased linearly with pain intensity only in the beginning of the experiment and subsequently habituated. In contrast, when the dopaminergic system was blocked by haloperidol, a linear increase with pain intensity was present throughout the entire experiment. Finally, there were no adaptive coding effects in any brain regions. Together, our findings provide novel insights into the nature of pain processing by suggesting that dopaminergic neurotransmission plays a specific role for the habituation to painful stimuli over time.

## Introduction

The accurate perception of nociceptive information, such as an electric shock, is crucial for survival and depends on the absolute intensity of the stimulus. For instance, increasing the magnitude of aversive stimulation leads to increased pain perception as well as increased neural activity in pain responsive brain regions, including somatosensory cortices, insular cortex and mid/anterior cingulate cortex (ACC; for a review see Iannetti and Mouraux, 2010). Although such *absolute coding* has frequently been reported, the processing of aversive information also depends on contextual factors. Specifically, neural responses to nociceptive stimuli decrease as a function of repetition. This is known as *habituation* (Glaser and Whittow, 1953). This habituation effect in response to electro-dermal stimulation was observed in the above-mentioned pain associated brain regions over consecutive experiment blocks, as shown with fMRI (Bingel et al., 2007; Mobascher et al., 2010) and simultaneous EEG-fMRI (Christmann et al., 2007). Similarly, in an fMRI study, Nickel et al. (2014) observed habituation to electrical stimuli in the secondary somatosensory cortex, insula, ACC, dorsolateral prefrontal cortex and inferior parietal lobule. In line with these neural effects, repeated exposure of the same painful stimulus, including heat, leads to decreased pain ratings (May et al., 2012).

While neural habituation to pain appears to be a protective mechanism in healthy humans, dysfunctional habituation may play a role in the chronification of pain (e.g., Bingel et al., 2007; Rodriguez-Raecke et al., 2014). Indeed, studies in chronic pain patients, for instance with chronic low back pain, migraine or fibromyalgia, showed attenuated habituation to pain when compared to healthy controls (e.g., Peters et al., 1989; Schoenen et al., 1995; de Tommaso et al., 2011). Importantly, several chronic pain pathologies such as fibromyalgia or burning mouth syndrome have been associated with dopaminergic deficits (e.g., Brefel-Courbon et al., 2005; Wood et al., 2007; Potvin et al., 2009; de Tommaso et al., 2011; Jarcho et al., 2012) indicating a link between habituation to pain and dopaminergic neurotransmission. For instance, patients with Parkinson’s disease, which is mainly characterized by a deficit of dopamine, showed attenuated habituation to nociceptive stimulation while they were off medication, while habituation was evident when dopamine was increased by levodopa treatment (dopamine precursor; Brefel-Courbon et al., 2005; Schestatsky et al., 2007). Moreover, formalin-induced nociception can be enhanced through the injection of D2 antagonists into the dorsolateral striatum (Magnusson and Fisher, 2000), and the dopaminergic substantia nigra/ventral tegmental area (SN/VTA) responds to painful stimuli as a function of probability (e.g., in animals: Matsumoto and Hikosaka, 2009; Bromberg-Martin et al., 2010; in humans: Bauch et al., 2014; Pauli et al., 2015) further suggesting a role of dopamine in pain processing.

Apart from habituation, contextual predictions also modulate pain processing. For instance, in direct comparison a high intensity electrical stimulation is perceived as more painful than a low intensity electrical stimulation (i.e., absolute coding); however, when high intensity stimulation is expected but the low one is delivered, it may be perceived as less intense. At the neural level, this can be related to neural adaptation which is a general property of neurons (Ohzawa et al., 1982; Brenner et al., 2000; Fairhall et al., 2001; Brown et al., 2008), and, more specifically, to the prediction error mechanism, which quantifies the difference between expected and received outcomes. Importantly, prediction errors adaptively scale according to the expected range of momentarily possible outcomes, which allows neurons to maintain high sensitivity. This neural mechanisms is well established in the reward literature (see below) and known as “adaptive coding” (e.g., Tobler et al., 2005; Bunzeck et al., 2010; Kobayashi et al., 2010; Park et al., 2012; Diederen et al., 2017).

For instance, in a single cell recording study in monkeys (Tobler et al., 2005), visual cues were followed with equal probability by either a small or medium reward (i.e., outcome), and another cue was associated with an upcoming large or medium reward. In both contexts, the larger of the two possible reward outcomes (i.e., medium and large) increased activity in the SN/VTA, while the relatively smaller reward led to activity decreases (i.e., scaled prediction errors). Similar forms of adaptive coding have been observed in the human ventral striatum, prefrontal cortex and medial temporal lobe (Tremblay and Schultz, 1999; Nieuwenhuis et al., 2005; Padoa-Schioppa and Assad, 2008; Padoa-Schioppa, 2009; Bunzeck et al., 2010). Moreover, there is evidence that dopamine modulates adaptive coding of appetitive information in the human midbrain and striatum (Diederen et al., 2017), which seem to be functionally connected (Park et al., 2012).

With regard to pain processing, several imaging studies have shown that contextual predictions (similar to adaptive coding) modulate activity in higher order pain-associated brain regions, including the insula and ACC (Wiech and Tracey, 2009; Ploner et al., 2011; Leknes et al., 2013; for a different finding see Winston et al., 2014). In particular, Leknes et al. (2013) presented within a block-design moderately painful heat stimuli either in a context with intense pain (i.e., relief context) or in a context with non-painful warm stimulation (i.e., control context). Both contexts were predicted by a unique cue; as such the moderately painful heat stimulus could be the best outcome (i.e., relief context) or the worst outcome (i.e., control context). In line with the literature on adaptive reward processing, pleasantness ratings were dependent on the context (called “hedonic flip” by the authors), and skin conductance as well as hemodynamic responses in the insula and dorsal anterior cingulate were higher in the control context as compared to the relief context. Although this activity pattern provides initial evidence for adaptive coding, the link to dopaminergic neuromodulation has not been demonstrated.

In sum, there is independent empirical evidence for: (a) absolute and (b) adaptive coding of painful stimuli, and for (c) neural habituation in pain responsive brain regions. However, the relationship to dopaminergic neurotransmission in healthy human subjects remains unclear. Therefore, we used a double-blind within-subject pharmacological fMRI study (placebo/haloperidol: dopamine antagonist) to investigated the role of dopamine in pain processing with a focus on absolute coding, adaptive coding and neural habituation.

To this end, the experiment consisted of two absolute tasks (phase I and phase III), where subjects rated the absolute intensity of an electro-tactile stimulus (low, medium, high) received on the back of the hand. Between these two phases, we used a task in which contextual predictions to a nociceptive event were manipulated across trials (i.e., adaptive task, phase II). Here, volunteers were presented with three different visual cues (i.e., triangle, square, diamond) predicting: (1) a high or medium electro-tactile stimulus; (2) a medium or low electro-tactile stimulus; or (3) a high or medium electro-tactile stimulus. We hypothesized neural habituation as well as adaptive coding in pain-associated areas, including the insula and ACC (Leknes et al., 2013), prefrontal cortex and possibly mesolimbic brain regions (SN/VTA, ventral striatum; Bunzeck et al., 2010; Leknes et al., 2013; Winston et al., 2014; Diederen et al., 2016). Moreover, we expected that neural habituation to absolute pain magnitude and adaptive coding diminishes in dopaminergic and pain-associated brain regions, when blocking dopaminergic D2 receptors with haloperidol.

## Materials and Methods

### Participants

Twenty-six participants took part in the fMRI experiment, but only 20 were included in further analyses (mean age: 25 years; age range: 19–28 years; 13 women) due to technical problems with the scanner or digitimer (electrical stimulation). All participants were healthy, right-handed and had normal or corrected-to-normal vision, without a history of neurological, psychiatric, or medical disorders or any current medical problems. This study was carried out in accordance with the recommendations of “Ethikkomission der Ärtzekammer Hamburg” with written informed consent from all subjects.

### Task

In order to assess dopaminergic effects on pain processing, a randomized double-blind within-subject design (i.e., 1.5 mg haloperidol/placebo) was used. Haloperidol is an established substance to treat psychiatric disorders including schizophrenia. More specifically, haloperidol is a dopaminergic antagonist that blocks mainly D2 receptors (e.g., Kapur et al., 1997). In correspondence with previous research, our dose of haloperidol has been shown to be an acceptable compromise between sufficient D2 receptor inhibition (between 60% and 80%) and minimization of side effects (Kapur et al., 1997).

**Figure 1:**
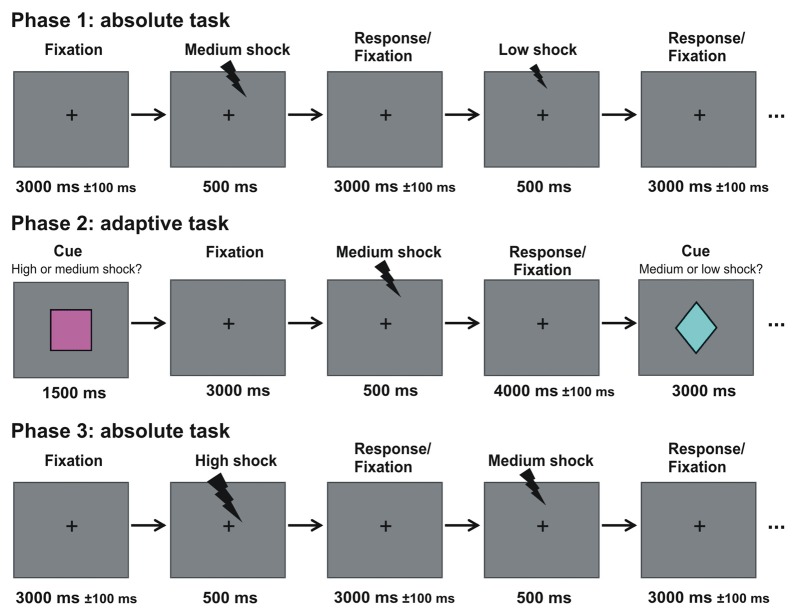
Experimental design. Participants underwent three phases. In the first and third phase (absolute task), electrical stimulation with three different magnitudes were presented randomly intermixed to the back of the hand. Subjects had to make low/medium/high judgments (see experimental procedures for details). In between both phases, participants took part in the adaptive task, where they learned the association between three contexts and three visual cues that predicted with 50% probability either: (1) a high or a medium stimulation; (2) a medium or a low stimulation; or (3) a high or a low stimulation. Participants made a relative low/high judgment (see experimental procedures for details).

The entire experiment took place in 3 days. During the first day, participants practiced a condensed version of the experiment to ensure high performance in all tasks, which will be explained in more detail below (see Figure 1). Furthermore, they received information regarding the MRI procedure and pharmacological manipulation. Participants were assigned to receive placebo on day 2 and haloperidol on day 3 or vice versa in a randomized and double-blinded fashion. The time interval between placebo-day and haloperidol-day was at least 7 days long (mean interval: 15 days) to allow a washout of haloperidol.

The experimental procedure was identical on both days of the pharmacological manipulation. Drug intake was scheduled 2.5 h before the start of the first phase in the MRI scanner, when haloperidol reaches significant plasma concentration (e.g., Andreou et al., 2014), and participants were asked to not eat 2 h before drug intake. Blood pressure was measured and a questionnaire about their subjective state and possible side effects was completed at three time points: before drug intake, 2.5 h after drug intake and after the experiment (i.e., approximately 4.5 h after drug intake). After placing the participant into the MRI scanner, an electrode was placed on the back of their right hand, which was followed by calibrating the magnitude for the highest possible electrical stimulation that was applied during the experiment.

Three different stimulation magnitudes were used during the experiment: low, medium and high. The stimulation magnitude was adjusted by varying the number of trains of consecutive electrical pulses of 2 ms each. Electrical stimulation was applied for 500 ms in total (i.e., high stimulation: 10 trains of 2 ms electrical pulses separated by an interval of 50 ms; medium stimulation: six trains of 2 ms pulses separated by an interval of 83.3 ms; low stimulation: four trains of 2 ms pulses separated by an interval of 125 ms). The time window between the first pulse and offset of the last pulse was identical for all shock intensities. This procedure was based on several published studies using the same electro-tactile stimulation system (Haaker et al., 2013; Lonsdorf et al., 2014; Sjouwerman et al., 2015). During calibration, participants were asked to rate the intensity of the electrical stimulation with 10 trains of electrical pulses on a visual analog scale (VAS) ranging from 0 (i.e., electrical stimulation is not perceptible) to 10 (i.e., electrical stimulation is intolerable). An intensity of seven for the electrical stimulation was used as highest possible nociceptive stimulus throughout the experiment. The magnitude of the medium and low stimulation conditions were adjusted accordingly by reducing the number of trains of pulses to six or four, respectively.

The experiment consisted of three consecutive phases: an absolute task (phase 1); an adaptive task (phase 2); and again an absolute task (phase 3; see Figure 1). The visual stimuli were presented via a mirror system attached to the head coil of the scanner. Throughout the first phase (absolute task), a fixation cross was presented in the center of a gray screen. A series of in total 60 consecutive electrical stimulations was applied to the back of the right hand. The three stimulation magnitudes were randomly intermixed (20 trials per condition) and each stimulation was separated by an inter-stimulus interval of 3000 ± 100 ms. Participants were asked to judge the magnitude of the electrical stimulation (i.e., low, medium, high) by pressing one of three buttons. The contingency between magnitude and button was randomly assigned across participants. Accuracy and speed were stressed. This phase took approximately 5 min in total. Ten practice trials were shown before the actual task to ensure that participants familiarized themselves with this task.

The second phase included a modified version of an established paradigm used in reward studies investigating adaptive coding (Tobler et al., 2005; Bunzeck et al., 2010; Park et al., 2012; Diederen et al., 2017). During this second phase (i.e., adaptive task), three visual cues (60 triangles, 60 squares; 60 diamonds; see Figure 1) were randomly intermixed and presented in central vision for 1500 ms on a gray background. Each cue was followed by a fixation cross that was shown throughout the remaining trial. Three-thousand millisecond after the offset of the cue, an electrical stimulation was applied for 500 ms. The three types of cues were associated with different contexts and predicted the occurrence of an electrical stimulation with either: (1) a high or medium; (2) a medium or low; and (3) a high or low stimulation intensity, whereby both stimulation intensities had the same occurrence probability in each type of context (e.g., in context 2, 50% of the stimulations had a medium and 50% a low intensity). The fixation cross disappeared 4000 ± 100 ms after stimulation offset. Note, that this paradigm is an event-related design.

On the first day (see above), all volunteers explicitly learned the association between the type of visual cue and stimulation magnitude in the behavioral lab outside of the scanner. The task was to judge whether the relatively low or high electrical stimulation was applied by pressing one of two buttons using the right index or middle finger, respectively. The response buttons were randomized across participants. Before the actual task in the scanner on day 2 and 3, participants were familiarized again with the task in a short practice consisting of 10 trials and accuracy and speed was stressed. The adaptive task was split into four blocks of 45 trials, and took approximately ~40 min including breaks.

Approximately 5 min after phase two ended, participants continued with the absolute task (i.e., phase 3) consisting of a different trial randomization than in phase 1. Finally, structural scans were acquired for approximately 15 min (see below).

Note that the relatively short jitters of 100 ms (Figure 1) might be longer in order to significantly improve design efficiency and should be adjusted in future experiments.

### fMRI Data Acquisition

The fMRI acquisition was performed on a 3-tesla system (Siemens Trio) with echo planar imaging (EPI). During functional imaging, 48 T2*-weighted images per volume (i.e., covering whole head) with BOLD contrast were obtained (matrix, 64 × 64; 48 oblique axial slices per volume angled at −30° along the anteroposterior axis; spatial resolution: 2 × 2 × 2 mm; TR = 2870 ms). For each subject, functional MRI data were acquired for both absolute tasks each consisting of 140 volumes and the adaptive task that was split into four scanning sessions each consisting of 148 volumes per session. Six additional volumes per scanning session were recorded at the beginning of each block to allow for steady-state magnetization; these were excluded from the analyses. At the end of the experiment, anatomical images of each subject’s brain were collected using multi-echo three-dimensional fast low angle shot (FLASH) acquisition for mapping T1 (TR = 19 ms), and magnetization transfer (TR = 24 ms) at 1-mm^3^ resolution (Weiskopf and Helms, 2008; Steiger et al., 2016).

### fMRI Data Analysis

All fMRI images were realigned to the first volume, unwarped, spatially normalized to the Montreal Neurology Institute space, and smoothed with a 4 mm Gaussian kernel using SPM12 (Ashburner et al., 2014). The fMRI time series data were high-pass filtered (cutoff = 128 s) and whitened using an AR(1) model.

For each subject, we computed four first-level analyses by including each combination between pharmacological treatment and task (i.e., (1) placebo and adaptive task; (2) placebo and absolute task; (3) haloperidol and adaptive task; and (4) haloperidol and absolute task). First, we defined four regressors for the absolute task under placebo treatment: one regressor for each stimulation magnitude (i.e., low, medium and high), and one regressor for trials with incorrect responses (i.e., errors). Second, we computed a first-level analysis for the same task under haloperidol treatment, which included the same four types of regressors as in the former analysis. A third first-level analysis was computed for the adaptive task under placebo. Here, we defined 10 regressors: one regressor for each of the three cues (i.e., (1) high/medium; (2) medium/low; and (3) high/low); one regressor for each of the six possible stimulation outcome (i.e., (1) high stimulation in context 1; (2) medium stimulation in context 1; (3) medium stimulation in context 2; (4) low stimulation in context 2; (5) high stimulation in context 3; and (6) low stimulation in context 3), and one regressor for trials with incorrect responses (i.e., errors). Fourth, we computed a first-level analysis for the adaptive task under haloperidol including the same regressors as defined in the third analysis. Note that the order of the trials for the different stimulation magnitudes was fully randomized. To capture residual movement-related artifacts, six covariates were included (the three rigid-body translation and three rotations resulting from realignment) as regressors of no interest in all four models.

Two separate second-level random-effects analyses (i.e., for absolute task and adaptive task) were computed on the contrast images resulting from the four first-level analyses. In the first second-level model, the hemodynamic effects of stimulation magnitude were entered into a 2 × 2 × 3 way analysis of variance (ANOVA) with the factor drug (placebo/haloperidol), time (phase 1/phase 3) and the factor stimulation magnitude (low/medium/high) to test for absolute pain effects and the influence of dopamine across time.

Second, a 2 × 3 × 2 way ANOVA with the factor drug (placebo/haloperidol), stimulation context (context 1/context 2/context 3) and stimulation outcome (relatively low/relatively high) was computed to investigate adaptive pain effects and the impact of dopamine (see Figure 1). This enabled us to investigate main effects and their interactions. We were interested in brain regions associated with an adaptive coding effect irrespective of drug treatment, which was realized by contrast weights of “1” for all relatively high stimulations and “−1” for all relatively low stimulations (see “Results” section).

All contrasts were initially thresholded at *p* < 0.001 (uncorrected). Since we hypothesized *a priori* regions for the adaptive effect (i.e., insula and ACC: Leknes et al., 2013), we corrected for multiple comparisons using small volume correction (SVC; *p* < 0.05, family-wise error (FWE)-correction, *k* > 5 voxels). The masks for the regions of interest were defined using the WFU-Pickatlas (Maldjian et al., 2003). Otherwise, FWE was used as implemented in SPM12.

The sources of the effects were localized by overlaying the SPMs on a T1-weighted group image, which was generated by averaging all normalized T1-images, respectively (spatial resolution of 1 × 1 × 1 mm).

## Results

### Behavioral Results: Questionnaire Regarding Side Effects

To account for potential drug effects on subjective well-being (i.e., side effects: dry mouth, dry skin, blurred vision, lethargy, nausea, dizziness and headache), participants rated their subjective state on a 7-point Likert scale three times during the experiment (before drug administration, 2.5 h after drug intake and at the end of the experiment after ~4.5 h). Mean ratings across the seven symptoms for the three measurements and both drug treatments are displayed in Table 1. The 2 × 3 ANOVA on subjective well-being (mean rating across seven side effects) with drug (placebo/haloperidol) and time (before drug intake, after 2.5 h, at the end) revealed neither a main effect of drug nor an interaction between time and drug (all *p*’s > 0.531). The analysis resulted in a significant main effect of time (*F*_(1.82,43.67)_ = 5.64; *p* = 0.009). However, *post hoc* paired *t*-tests revealed no difference between the three time points when *p* values were Bonferroni corrected (all *p*’s > 0.113).

**Table 1:** Likert ratings for potential side effects (1 = no side effects; 7 = extreme side effects) before, during and after the fMRI experiment in the placebo and haloperidol condition (*n* = 20).

Treatment	1st assessment (before DI)	2nd assessment (2.5 h after DI)	3rd assessment (4.5 h after DI)
Placebo	1.54 (0.46)	1.43 (0.30)	1.44 (0.39)
Haloperidol	1.54 (0.44)	1.41 (0.39)	1.53 (0.42)

*Values represent mean ratings across-subject means (SD). DI, drug intake*.

During calibration, averaged intensity ratings for the highest electrical stimulation on the VAS from 0 (i.e., electrical stimulation is not perceptible) to 10 (i.e., electrical stimulation is intolerable) did not differ between placebo (*M* = 1.42 mA; SD = 0.45; range 1.42–2.3 mA) and haloperidol treatment (*M* = 1.45 mA; SD = 0.63; range 1.44–3.00 mA; *p* = 0.840).

### Behavioral Results: Absolute Task

Mean reaction times (RTs) are displayed in Table 2. A 2 × 3 × 2 way ANOVA with the factor drug (placebo/ haloperidol), stimulation magnitude (low/medium/high) and time (phase 1/phase 3) revealed a significant main effect of stimulation magnitude (*F*_(2,1.48)_ = 31.37; *p* < 0.0001). Mean RTs for medium stimulation were slower than for high (*t*_(19)_ = −3.76; Bonferroni corrected *p* = 0.009) and low stimulation (*t*_(19)_ = 8.50; Bonferroni corrected *p* = 0.003); and participants responded faster to high stimulation than to low stimulation (*t*_(19)_ = 4.17; Bonferroni corrected *p* = 0.009). Analyses showed no main effect of drug, time or an interaction between any of the factors (all *p*’s > 0.191).

**Table 2:** Reaction times (RTs) of hits in the absolute task for the first and third phase (*n* = 20).

Drug	Phase	Stimulation magnitude		
		Low	Medium	High
Placebo	I	1115 (193)	1384 (192)	1259 (180)
	III	1119 (206)	1373 (293)	1191 (168)
Haloperidol	I	1111 (301)	1465 (227)	1241 (377)
	III	1135 (172)	1390 (210)	1268 (257)

*Values represent RTs of correct responses across-subject means in ms (SD)*.

The mean proportion of hits for each condition is displayed in Table 3. A 2 × 3 × 2 way ANOVA on the mean hit rate revealed no main effect of stimulation magnitude, drug treatment and there was no interaction between any factors (all *p*’s > 0.081).

**Table 3:** Proportion of hits in the absolute task for the first and third phase (*n* = 20).

Drug	Phase	Stimulation magnitude		
		Low	Medium	High
Placebo	I	0.83 (0.11)	0.70 (0.14)	0.72 (0.21)
	III	0.76 (0.19)	0.74 (0.19)	0.77 (0.19)
Haloperidol	I	0.74 (0.24)	0.66 (0.15)	0.64 (0.22)
	III	0.76 (0.16)	0.75 (0.14)	0.68 (0.12)

*Values represent proportions of correct responses across-subject means (SD)*.

### Behavioral Results: Adaptive Task

The mean RTs are depicted in Table 4. A 2 × 3 × 2 ANOVA with the factor drug (placebo/haloperidol), context (1/2/3) and relative stimulation outcome (low/high) revealed a significant main effect of context (*F*_(1.91,40.03)_ = 44.03; *p* < 0.0001), stimulation outcome (*F*_(1,19)_ = 34.07; *p* < 0.0001) and a significant interaction between context and stimulation outcome (*F*_(1.94,36,84)_ = 85.49; *p* < 0.0001), but there was no effect of drug (*p*’s > 0.214). Mean RTs in response to the relatively lower stimulation were faster than to the high stimulation (see Table 4). Mean RTs in context 3 were significantly faster than RTs in context 1 and context 2, where the physical difference between the stimulation is relatively smaller. Mean RTs in context 1 and 2 did not differ. Mean RTs in response to the low stimulation magnitude in both contexts (i.e., context 2: *t*_(19)_ = 10.53; *p* < 0.0001); context 3: (*t*_(19)_ = 3.42; Bonferroni corrected *p* = 0.009; other *p* = 0.107) were faster in comparison to the response to the relatively high stimulation outcome. There was no difference in mean RTs between the medium and high stimulation (i.e., context 1).

**Table 4:** RTs of hits in the adaptive task (*n* = 20).

Drug	Context	Relative stimulation magnitude	
		Low	High
Placebo	1 (med/high)	1276 (243)	1272 (272)
	2 (low/med)	1104 (145)	1447 (212)
	3 (low/high)	1041 (161)	1119 (186)
Haloperidol	1 (med/high)	1377 (243)	1289 (267)
	2 (low/med)	1127 (147)	1489 (254)
	3 (low/high)	1070 (219)	1158 (221)

*Values represent RTs of correct responses across-subject means in ms (SD)*.

The proportion of correct responses is summarized in Table 5. A 2 × 3 × 2 ANOVA revealed a significant main effect of context (*F*_(1.79,34.07)_ = 75.65; *p* < 0.0001) and a significant interaction between context and stimulation outcome (*F*_(1.34 25.54)_ = 4.09; *p* = 0.043), but no interaction with the factor drug (other *p*’s > 0.089). Separate *post hoc* *t*-tests for each context revealed no difference between both stimulation outcomes (all *p*’s > 0.093).

**Table 5:** Proportion of hits in the adaptive task (*n* = 20).

Drug	Context	Relative stimulation magnitude	
		Low	High
Placebo	1 (med/high)	0.90 (0.09)	0.87 (0.12)
	2 (low/med)	0.86 (0.12)	0.78 (0.18)
	3 (low/high)	0.96 (0.06)	0.96 (0.08)
Haloperidol	1 (med/high)	0.86 (0.12)	0.87 (0.10)
	2 (low/med)	0.87 (0.10)	0.75 (0.17)
	3 (low/high)	0.93 (0.10)	0.93 (0.08)

*Values represent proportions of correct responses across-subject means (SD)*.

### Imaging Results for Absolute Task (Phase 1 and 3)

**Figure 2:**
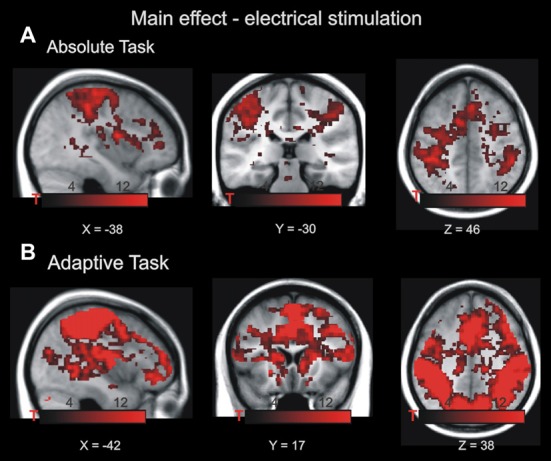
Main effect of electrical stimulation in the absolute task **(A)** and adaptive task **(B)**. The highlighted voxels exhibited increased BOLD activity during noxious stimulation *p* < 0.001, family-wise error (FWE)-corrected at cluster level. Maps of activations are superimposed on a T1 group template. Note that both tasks require different cognitive demands; therefore, both main effects are not formally compared.

Imaging data for the absolute task in phase 1 and 3 were analyzed in a 2 × 3 × 2 ANOVA with the factors drug (placebo/haloperidol), stimulation magnitude (low, medium, high) and time (phase 1/phase 3). In a first step, we aimed to pinpoint brain regions that were associated with the processing of pain irrespective of drug and stimulation magnitude across both phases and drug conditions (i.e., main effect of pain, Figure 2A and https://neurovault.org/images/57884/). Whole brain analyses revealed BOLD effects in left central operculum (−54 −18 22; 24981 voxels; FWE corrected *p* < 0.0001); right putamen (14 6 −10; 396 voxels; FWE corrected *p* < 0.0001); right angular gyrus (34 −62 50; 62 voxels; FWE corrected *p* = 0.014); and cerebellar vermal lobules I-V (4 −62 −12; 117 voxels; FWE corrected *p* < 0.0001). Although this activation pattern is widely distributed and rather unspecific in nature, it confirms previous studies on pain processing because it involves the expected brain regions (see “Introduction” section).

**Figure 3:**
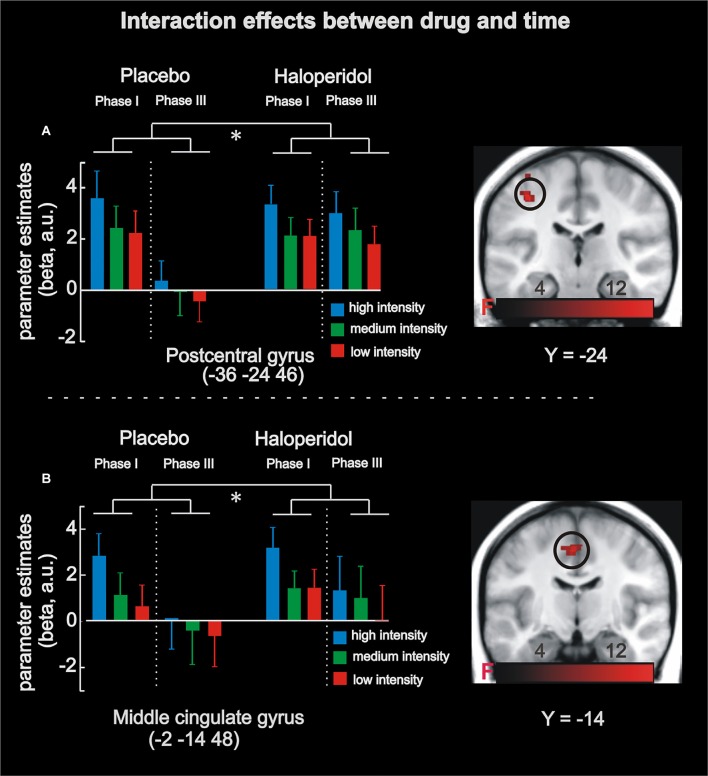
fMRI results in the absolute task. Analyses revealed a significant interaction between stimulation magnitude, drug and time in left postcentral gyrus **(A)** and left midcingulate cortex **(B)**. Under placebo in phase I, activity increased as a function of stimulation magnitude (absolute coding), and this effect was absent in phase III (i.e., habituation). Under haloperidol, however, there was no significant habituation from phase I to phase III. The significant interaction effects are highlighted by the asterisk. Maps of activations are superimposed on a T1 group template. Error-bars denote one standard error of the mean.

In a second step, the analysis revealed BOLD responses that linearly coded absolute stimulation magnitude irrespective of drug treatment (i.e., main effect of absolute coding) in the left midcingulate cortex supplementary motor cortex (−2 −14 48; 206 voxels; FWE-corrected *p* < 0.0001) and left postcentral gyrus (−38 −24 48; 422 voxels; FWE-corrected *p* < 0.0001). Finally, a significant 2 × 3 × 2 interaction effect was observed in the left postcentral gyrus (−36 −24 46; 123 voxels; FWE-corrected *p* < 0.0001) and the left midcingulate cortex (−2 −14 48; 91 voxels; FWE-corrected *p* < 0.0001), contralateral to the stimulated region. This effect was driven by a linear activity increase of stimulation magnitude in phase 1 for both drug conditions (placebo and haloperidol), and, importantly, this linear effect disappeared in phase 3 in the placebo but not haloperidol condition (see Figure 3). Thus, a time-dependent neural habituation to painful events (placebo) was blocked by haloperidol.

### Imaging Results for Adaptive Task

Imaging data for the adaptive task (phase 2) were analyzed in a 3 × 2 ANOVA with the factors drug (placebo/haloperidol), context (high/medium, medium/low, high/low). First, we were interested in pain-related activity independent of context and drug (i.e., main effect of pain, Figure 2B and https://neurovault.org/images/57885/). BOLD effects were evident in bilateral postcentral gyrus (−44 −22 56; 83447 voxels; FWE-corrected *p* < 0.0001; 64 −14 32; 1403 voxels; FWE-corrected *p* < 0.0001); left supplementary cortex/mid-cingulate cortex (−2 −6 50; 4075 voxels; FWE-corrected *p* < 0.0001); bilateral anterior insula (−42 −6 6; 582 voxels; FWE-corrected *p* < 0.0001; 44 −2 4; 587 voxels; FWE-corrected *p* < 0.0001). Similar to the main effect of pain in the absolute task, this activation pattern is widely distributed and rather unspecific in nature, but it confirms previous studies on pain processing (see “Introduction” section).

In the next step, we identified brain regions with adaptively coded responses to stimulation outcome irrespective of drug treatment (i.e., main effect of adaptive coding). FWE-corrected whole brain analysis did not reveal any significant effects. Subsequently, a SVC was performed, using the insula and ACC as masks (see “Introduction” section: Ploner et al., 2011; Leknes et al., 2013); this analysis also did not reveal any significant main effects or interactions (*p* < 0.05, FWE-corrected, *k* > 5 voxel).

## Discussion

### Summary of Main Findings

We investigated the neural mechanisms underlying pain processing with a focus on contextual effects and dopaminergic neurotransmission in a pharmacological fMRI experiment. Our data reveal that haloperidol decreased neural adaptation to electrical stimulation in pain-associated areas (Iannetti and Mouraux, 2010), including left postcentral gyrus and left midcingulate cortex. While this habituation effect was evident under placebo, it was absent after haloperidol intake (see Figure 3) suggesting a direct link to dopaminergic neurotransmission.

### fMRI Findings for Absolute Task

Absolute coding of pain magnitude in the left midcingulate cortex and left postcentral gyrus is consistent with previous fMRI studies reporting BOLD increases as a function of pain intensity in brain regions associated with pain or saliency processing. This includes primary and secondary somatosensory cortices, the insular cortex and mid/ACC (Coghill et al., 1999; Büchel et al., 2002; Iannetti and Mouraux, 2010). Specifically, the midcingulate cortex has been involved in cognitive control processes, preparation of defensive responses to threat (for review, see Shackman et al., 2011) and in discriminating between pain intensities (Büchel et al., 2002).

The habituation effect in the left postcentral gyrus and mid-cingulate cortex in the placebo group corresponds to previous research showing that habituation to nociceptive stimuli can already be evident within a short period of time (e.g., Milne et al., 1991). For instance, electro-dermal stimulation is associated with a reduction of BOLD responses from the first to the second half of the experiment in primary and secondary somatosensory cortices, the insular and anterior/mid cingulate cortex (Mobascher et al., 2010; see also Ibinson et al., 2004; Bingel et al., 2007; Christmann et al., 2007). Similar findings on habituation have been reported by Rennefeld et al. (2010) and Nickel et al. (2014). Also in line with previous literature, our effects were evident contralateral to the stimulated hand (e.g., Peyron et al., 2000; Bingel et al., 2003). However, in comparison to former habituation studies, we used three different stimulation magnitudes, which all showed habituation over time. The absence of a graded reduction of BOLD response for the three different pain magnitudes in the third phase (i.e., absence of an absolute effect) indicates that habituation to pain is rather an all-or none phenomenon.

Importantly, the interaction between drug and time provides evidence for the role of dopamine in pain processing. At the physiological level, haloperidol reduces dopamine availability by blocking dopamine D2 receptors (Kapur et al., 1997) and striatal dopamine D2 receptors are known to modulate pain processing (Hagelberg et al., 2004; Potvin et al., 2009). Thus, blocking dopaminergic neurotransmission may have prevented the habituation to electrical stimulation across time. Indeed, clinical studies suggest that habituation is reduced in chronic pain patients, such as fibromyalgia or migraine (Valeriani et al., 2003; Montoya et al., 2006; Smith et al., 2008), with a link to dopaminergic deficits (Potvin et al., 2009). For instance, Parkinson’s patients showed reduced neural and behavioral habituation to pain stimuli in the absence of levodopa treatment (i.e., dopaminergic stimulation; Schestatsky et al., 2007; see also Martikainen et al., 2015). Moreover, animal models stressed that chronic pain is associated with decreased D2 receptor availability and excitatory functions of D2 neurons in the nucleus accumbens (Schwartz et al., 2014). Finally, intraventricular or striatal microinjections of haloperidol increased acute pain and apomorphine (dopamine agonist) reduced nociception (Lin et al., 1981; Magnusson and Fisher, 2000; Mansikka et al., 2005). Thus, our findings provide evidence for the role of dopamine in pain processing by showing reduced neural habituation following receptor blockage in pain-associated brain regions.

### No fMRI Findings for Adaptive Task

Contrary to our predictions, adaptive coding was not evident in the mesolimbic system, including the ventral striatum (e.g., Bunzeck et al., 2010; Park et al., 2012) and dopaminergic SN/VTA (Tobler et al., 2005; Matsumoto and Hikosaka, 2009; Bauch et al., 2014; Diederen et al., 2016). One possibility for this null finding is that the spatial resolution of fMRI could have been too low to dissociate between subsets of SN/VTA neurons showing adaptive coding and others responding in an absolute fashion. Alternatively, adaptive coding within the dopaminergic system might depend on task properties. In fact, hemodynamic responses within the human SN/VTA and ventral striatum were adaptively coded in a paradigm where reward distributions alternated in short blocks (rather than trial wise) and had to be learned throughout the experiment (Diederen et al., 2016). This indicates that adaptive coding to appetitive and aversive information may be more pronounced in implicit and blocked learning paradigms. Together with higher spatial resolution, both aspects should be regarded in future studies. A third possibility is that adaptive coding of nociceptive events does not depend on the dopaminergic mesolimbic system but is driven by other neuromodulators such as the opioid or norepinephrine system (e.g., Wager et al., 2007; Scott et al., 2008).

For the insula and ACC, we also hypothesized adaptive coding effects on the basis of a study by Leknes et al. (2013), which could not be confirmed. Here, our null effects might be due to differences in the design (block vs. event related), or related to differences in sensory modality (heat vs. electrical stimuli). A final possibility is that adaptive coding is not as relevant to pain processing as it is for reward. Indeed, rewarding stimuli, such as monetary incentives, have a much wider range, which might require higher fidelity, while aversive stimuli on the other hand have a natural upper limit.

### Limitations

There was no behavioral effect of haloperidol in any task of the combined pharmacological fMRI study. Instead, participants were equally able to differentiate between stimulation intensities irrespective of time and drug. This suggests that dopamine does not influence the perceptual and discriminative processes of nociceptive information *per se*, but may indirectly modulate pain processing via higher cognitive functions, such as learning or valuation processes (see also Becker et al., 2013; Tiemann et al., 2014). Since we only sampled the discriminating performance between different pain magnitudes, it is an open question whether subjective pain ratings to the different electrical stimulation also habituate over time (e.g., Bingel et al., 2007; Mobascher et al., 2010) and vary as a function of haloperidol. Alternatively, the effect of haloperidol on neural processes but not behavior may be due to the relatively low single dosage. Indeed, similar reports (i.e., no effects of drug on behavior) have been published in the field of placebo research (Wrobel et al., 2014) and pain sensitivity (D2 antagonist sulpiride, Becker et al., 2013).

As a final remark, we would like to point out that future research needs to include other non-nociceptive types of stimuli to investigate whether the dopaminergic effect on habituation is specific to pain processing.

## Conclusion

Haloperidol changed the habituation to painful events over time in left postcentral gyrus and left midcingulate cortex. As such, our results point towards a previously unreported mechanism linking dopaminergic neuromodulation and habituation to pain in healthy humans.

## Author Contributions

EMB, VHR and NB designed research. EMB and CA performed fMRI data acquisition. EMB, NB, CA and VHR interpreted the data. EMB and NB drafted the article. CA and VHR revised the article critically for important intellectual content. EMB, CA, VHR and NB gave final approval for the article to be published; agreed to be accountable for all aspects of the work.

## Conflict of Interest Statement

The authors declare that the research was conducted in the absence of any commercial or financial relationships that could be construed as a potential conflict of interest.
